# Molecular mechanisms of severe acute respiratory syndrome (SARS)

**DOI:** 10.1186/1465-9921-6-8

**Published:** 2005-01-20

**Authors:** David A Groneberg, Rolf Hilgenfeld, Peter Zabel

**Affiliations:** 1 Pneumology and Immunology, Otto-Heubner-Centre, Charité School of Medicine, Free University and Humboldt-University, D-13353 Berlin, Germany; 2 Institute of Biochemistry, University of Lübeck, D-23538 Lübeck, Germany; 3 Division of Clinical Infectiology and Immunology, Department of Medicine, Research Center Borstel, D-23845 Borstel, Germany; 4 Division of Thoracic Medicine, Department of Medicine, University of Lübeck, D-23538 Lübeck, Germany

**Keywords:** Severe Acute Respiratory Syndrome, SARS, coronavirus, molecular mechanisms, therapy, vaccination

## Abstract

Severe acute respiratory syndrome (SARS) is a new infectious disease caused by a novel coronavirus that leads to deleterious pulmonary pathological features. Due to its high morbidity and mortality and widespread occurrence, SARS has evolved as an important respiratory disease which may be encountered everywhere in the world. The virus was identified as the causative agent of SARS due to the efforts of a WHO-led laboratory network. The potential mutability of the SARS-CoV genome may lead to new SARS outbreaks and several regions of the viral genomes open reading frames have been identified which may contribute to the severe virulence of the virus. With regard to the pathogenesis of SARS, several mechanisms involving both direct effects on target cells and indirect effects via the immune system may exist. Vaccination would offer the most attractive approach to prevent new epidemics of SARS, but the development of vaccines is difficult due to missing data on the role of immune system-virus interactions and the potential mutability of the virus. Even in a situation of no new infections, SARS remains a major health hazard, as new epidemics may arise. Therefore, further experimental and clinical research is required to control the disease.

## Introduction

Severe acute respiratory syndrome (SARS) is the first new infectious disease of this millennium. SARS has originated from Southern China at the end of 2002 and has a high mortality and morbidity. Within a period of six months beginning at the end of 2002, the disease has affected more than 8,000 people and killed nearly 800 [1]. The disease poses a new threat for respiratory medicine and represents a challenge for antiviral drug development and administration [2,3].

SARS is caused by a novel, SARS-associated coronavirus (SARS-CoV) [4-6] which has been identified by a World Health Organization (WHO)-led global laboratory network. The first cases of SARS were reported from a hospital in Hanoi, Vietnam, by Carlo Urbani, a WHO scientist who himself died from the disease [7]. After reports from health authorities in Hong Kong on the outbreak of a new form of epidemical atypical pneumonia in public hospitals, the WHO issued a global alert on the disease. During this period, cases of SARS were also reported from China, other Asian countries and even other continents including America (Canada, U.S.A.) and Europe (Germany).

Shortly after the initial global alert, the WHO initiated a collaborative multi-center research project on SARS diagnosis, led by eleven principal laboratories in nine countries [8]. Using modern communication technologies to optimize the analysis of SARS tissue samples, it was soon shown that a novel coronavirus is the causative agent of SARS (SARS-CoV) [4-6]. Due to the death of Carlo Urbani who first identified the new disease, the first isolate of the virus was proposed to be named Urbani strain of SARS-associated coronavirus, but a final terminology has not been proposed so far [9]. Since Koch's principles have been shown to be fulfilled by the new pathogen [10,11], it is not necessary to call the virus SARS-associated and the general agreement is now to call it SARS coronavirus (SARS-CoV).

Parallel to the progress made in the epidemiology and clinical diagnosis which has recently been demonstrated by numerous case reports, clinical studies and definitions [1], scientists have also revealed basic mechanisms of the underlying causative agent, the SARS coronavirus. As it is crucial for future strategies that SARS is detected in its earliest stages and that therapeutic options are optimized, insights into the molecular mechanism of SARS have to be used to develop new therapeutic strategies and vaccines.

While other reviews have focused on the epidemiology, clinical presentation and potential treatment of SARS, the present overview aims to analyze and present the currently available data on molecular mechanisms of SARS. In this respect, it is important to underline that in the present state of no specific drug or vaccine being available, research on molecular mechanism is crucial to identify potential treatment targets.

### Etiology

Prior to the development of therapeutic regimes based on molecular mechanisms of the disease, the causative agent had to be isolated and analysed. Soon after the fast establishment of the international WHO laboratory network, rapid progress was made in the identification process of the causative agent, and it was reported that SARS is most probably caused by a novel strain of the family of coronaviruses [4-6]. These viruses are commonly known to cause respiratory and gastrointestinal diseases of humans and domestic animals [12,13]. The group of coronaviruses is classified as a member of the order nidovirales, which represents a group of enveloped positive-sense RNA viruses consisting of coronaviridae and arteriviridae [14]. Viruses of this group are known to synthesize a 3' co-terminal set of subgenomic mRNAs in the infected cells [15].

### Origin of the SARS virus

Soon after the identification of a new coronavirus as the causative agent of SARS and of a southern Chinese province as the first area of occurrence, animal species of this area have been speculated to be the origin of the SARS-CoV. As analysis of the SARS-CoV genetic sequence revealed large differences to any other currently known coronaviruses in humans or domestic animals [16,17], it was hypothesized that the new virus might originate from wild animals. This hypothesis was supported by a search for coronaviruses in wild animals sold on markets in southern China, which identified the presence of a coronavirus in civet cats. This animal coronavirus was shown to have a sequence identity of more than 99% to the SARS coronavirus [18] with only a limited number of deletions and mutations between both viruses. SARS-CoV has a deletion of 29 nucleotides relative to the civet cat virus, indicating that if there was direct transmission, it went from the animal to man, because deletions occur probably more easily than insertions. Recent reports indicate that SARS-CoV is distinct from the civet cat virus and it has not been answered so far if the civet cat virus is the origin of the SARS-CoV or if civet cats were also infected from other species [19]. Therefore, there are no data available on the possibility of horizontal transmission between animals, and the question whether the jump of the virus from an animal to humans was a single accident or may frequently occur in future with the animals as dangerous reservoirs for future SARS epidemics remains unanswered. So far, the SARS-CoV has been reported to be able to infect not only humans but also macaque monkeys [11], domestic cats, and ferrets [20]. However, transmission of the virus from the domestic cat to man has not been shown. The ability of the SARS-CoV to infect other animal species could point to potential natural reservoirs of the virus. In this respect, coronaviruses are known to relatively easily jump to other species. I.e., the human coronavirus OC43 shares a high degree of genetic sequence homology to bovine coronavirus (BCoV) and it is commonly assumed that it has jumped from one species to the other [21,22]. In the same way, BCoV has been reported to be able to infect humans and cause diarrhea [23]. Whereas the precise mechanisms of these species jumps remain unclear, it is most likely that they represent the results of mutations and epidemiological studies of coronavirus infections in wild animals will therefore be crucial for future understanding and control of new SARS outbreaks.

### SARS virus taxonomy

**Figure 1:**
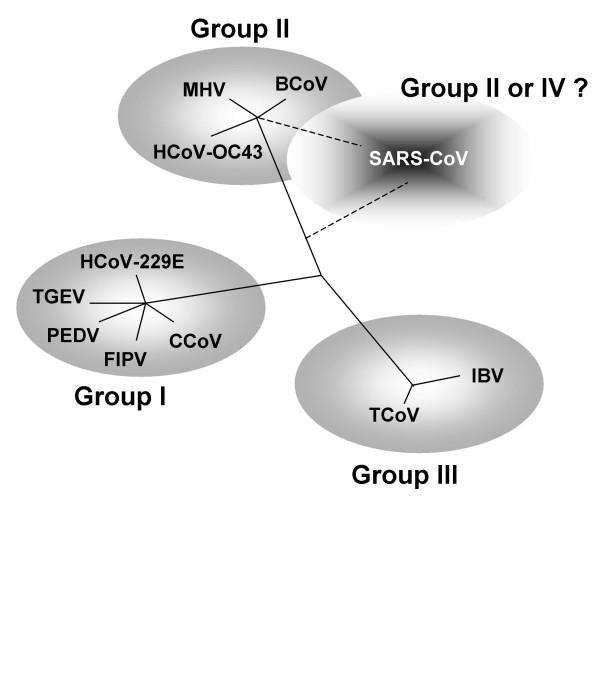
Coronavirus classification. The family of coronaviruses belongs to the order of nidovirales and consists of three groups so far. It is still debatable whether the new SARS-CoV should be assigned to group II or to a new fourth group. Group I includes human coronavirus 229E (HCoV-229E), transmissible gastroenteritis virus (TEGV), porcine epidemic diarrhea virus (PEDV), canine coronavirus (CCoV), and feline coronavirus (FIPV). Group II viruses include human coronavirus OC43 (HCoV-OC43), murine hepatitis virus (MHV), and bovine coronavirus (BCoV), and group III species are turkey coronavirus (TCoV), and avian infectious bronchitis virus (IBV).

Until the identification of the new SARS-CoV, the coronaviruses have been divided into three subgroups, which differ with respect to their genome [24]. The first group consists of viruses such as the human coronavirus 229E (HCoV-229E), porcine respiratory coronavirus (PRCV), porcine transmissible gastroenteritis virus (TGEV), feline infectious peritonitis virus (FIPV) and feline enteritis virus (FEV) or the canine coronavirus (CCoV). The second group comprises human coronavirus OC43 (HCoV-OC43), bovine coronavirus (BCoV), and mouse hepatitis virus (MHV), and the third group mainly consists of avian species such as the chicken infectious bronchitis virus (IBV). Whereas the SARS-CoV has been shown to cross-react with some group I coronavirus antibodies [6], its genetic sequence does not belong to this group. Within the nucleic acid or protein sequence phylogenetic trees of the coronavirus family, the SARS-CoV has first been located at an equal distance from the second and third group, irrespective of which SARS-CoV RNA region is used for analysis [6,16,17]. Therefore, the SARS-CoV may represent the first member of a new group of coronaviruses (Figure 1). However, the taxonomy is still no clear [19,25], and recent studies that focused on the N-terminal domain of the spike protein and on poorly conserved proteins such as Nsp1, matrix protein, or nucleocapsid, have suggested a relation to group II viruses [26]. A similar conclusion can be drawn if the polymerase gene is examined, pointing to an early split-off from the coronavirus group 2 lineage [27].

Despite the fact that this new virus most likely jumped to humans from wild animal species, it has remarkably well adapted to the human organism as shown by its high person-to-person transmissibility.

### SARS virus genome structure

The structure of the SARS viral RNA is organized in 13–15 open reading frames (ORF) and contains a total of approximately 30,000 nucleotides [6,16,17].

Recently, 61 SARS-CoV sequences derived from the early, middle, and late phases of the SARS epidemic together with two viral sequences from palm civets were analyzed [28]. Genotypes characteristic of each phase were discovered, and it was found that the neutral mutation rate of the viral genome was constant but the amino acid substitution rate of the coding sequences slowed during the course of the epidemic. The spike protein showed the strongest initial responses to positive selection pressures [28].

Only ORFs exceeding fifty amino acids in translational capacity are considered relevant as they contain the sequences for the structural and functional properties of the virus and are therefore of potential interest for the development for future therapeutic strategies. The comparison of the different SARS-CoV ORFs with those of other coronaviruses reveals a familiar pattern of structural gene arrangement with replicase and protease genes (gene 1a-1b) and the spike (S), envelope (E), membrane (M) and nucleocapsid (N) genes in a typical 5'- to 3' order of appearance [29]. The proteins encoded by these genes may be targets for novel treatments. Between these well-known genes, a series of ORFs of unknown function was found: There are two ORFs situated between the spike and the envelope genes and three to five ORFs between the membrane and nucleocapsid genes. Comparison of this gene organization with other known coronaviruses does not indicate a closest proximity to group II coronaviruses. Also, the SARS-CoV genomic sequence does not contain a gene for hemagglutinin-esterase (HE) protein, which is present in the majority of group II coronaviruses.

**Figure 2:**
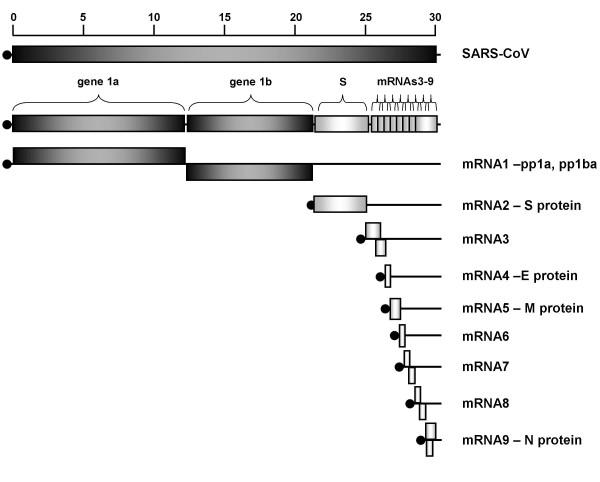
SARS-CoV genome organization. The structure of the SARS viral RNA is organized into 13–15 open reading frames (ORFs) and contains an overall amount of approximately 30,000 nucleotides. The sequence can be separated into different elements and genomic and subgenomic mRNAs.

Two-thirds of the SARS RNA is organized in the gene 1a-1b. The sequence of this gene is highly conserved among all coronaviruses [17]. ORFs 1a and 1b encode two polyproteins, pp1a and pp1ab, the latter through a ribosomal frameshifting mechanism. These polyproteins are processed by virus-encoded proteinases, to yield 16 individual proteins. Most potential gene 1a-1b products are fairly well conserved between SARS-CoV and other coronaviruses [17,29]. Many of their functions are unknown but it is suggested that they participate in viral RNA replication, making them potential targets for the development of antiviral compounds. Therefore, research efforts will focus on these proteins. One exception from the overall conservation of SARS-CoV gene 1a-1b is the lack of a sequence coding for PL1^pro^, one of the two papain-like proteinases operating on cleavage sites at the N-terminus of the polyproteins (Figure 2). The main proteinase (M^pro^), also called 3C-like protease (3CL^pro^), is responsible for the cleavage of all the remaining proteins encoded by gene 1a-1b [29,30].

### SARS virus gene expression

Apart from gene 1, coronavirus genes are known to be usually expressed from subgenomic mRNAs. They share a common leader sequence at the 5'-end and initiate at different places in the genome extending toward the 3'-end of the virus genome [31]. Some ORFs may also be unconventionally translated from a single mRNA. As these uncommon translation mechanisms are not very efficient and the gene products are not very abundant, these ORFs typically encode nonstructural proteins. Whereas the ORFs between the structural protein genes are very heterogeneous among the different coronaviruses and not essential for viral replication, recent studies suggested that deletion of non-essential ORFs may result in a reduced virulence [32]. In agreement with this, some of these non-essential ORFs of the new SARS-CoV genome may be responsible for the high SARS-CoV virulence.

So far, five to eight subgenomic mRNAs were found in SARS-CoV-infected cells [17,27]. Thiel and colleagues performed the first detailed study on mechanisms and enzymes involved in SARS-CoV genome expression (Figure 2) [29]. They determined the sequence of the SARS-CoV isolate Frankfurt 1 and characterized the major RNA elements and protein functions involved in the genome expression by characterizing regulatory mechanisms such as the discontinuous synthesis of eight subgenomic mRNAs, ribosomal frameshifting and post-translational proteolytic processing. Also, the activities of SARS-CoV enzymes such as the helicase or the two cysteine proteinases (PL2^pro ^and M^pro^) were addressed as they are involved in replication, transcription or post-translational polyprotein processing [29].

In conclusion, research in the area of coronavirus gene expression is important to delineate components which directly affect SARS-CoV virulence.

### SARS virus structural proteins

The structural proteins of the new SARS-CoV are potential targets for new treatment options. The new SARS-CoV only contains the three envelope proteins, spike (S), envelope (E), and membrane (M) but not the hemagglutinin-esterase (HE) protein, which is present in some coronaviruses of the second group.

**Figure 3:**
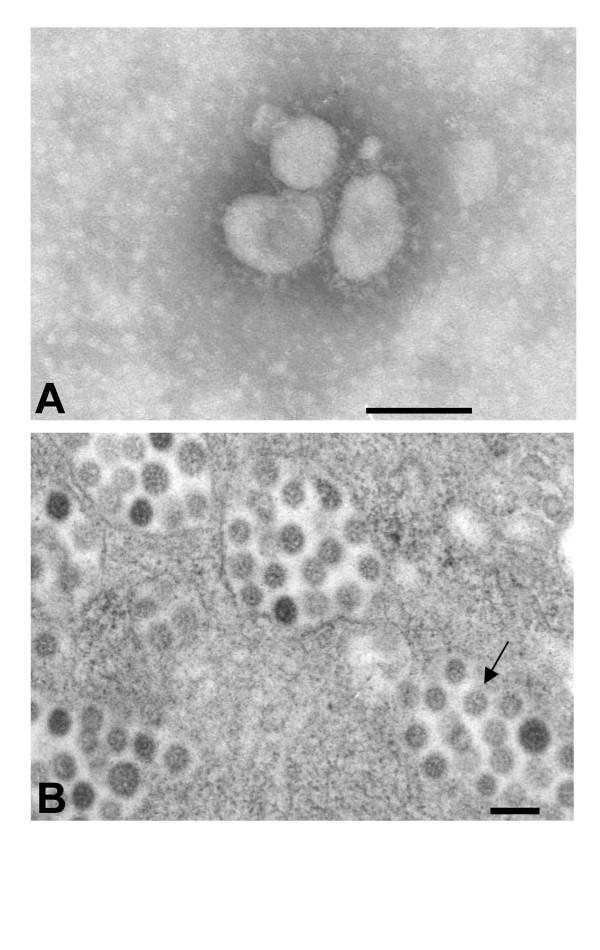
SARS-CoV transmission electron microscopy. In the supernatant of SARS-CoV infected cytopathic Vero E6 cells, characteristic virus particles can be found. The diameter of the viruses ranges between 60 nm and 120 nm and the virus shapes are round or oval. There are many protrusions from the envelope which are arranged in order with wide gaps between them. There are also many virus particles in the infected cells present. They often form a virus vesicle with an encircling membrane. A: Higher magnification B: Lower magnification. Scale bars represent 100 nm. Reproduced with permission from Acta Biochimica et Biophysica Sinica 2003, 35(6):587–591 [126].

The spike glycoprotein is responsible for the characteristic spikes of the SARS-CoV (Figure 3). Intra- and extracellular proteases often cleave the S protein into S1 and S2 domains, with the cleavage process often increasing infectivity of the virus. Molecular modelling has been performed for the S1 and S2 units of the SARS-CoV spike protein [33,34]. The spike proteins of coronaviruses are reported to bind to receptors on their target cells and the domains responsible for receptor-binding are commonly situated in the N-terminal region of S1 [35-40]. The spikes consist of oligomeric structures, that are formed by heptad repeats of the S2 domain which also represent a fusion peptide sequence. This peptide is responsible for the coronavirus fusion activity.

The SARS-CoV has also been reported to cause the formation of syncytia in vivo, but so far only under the condition of cultured Vero cells [6]. The SARS-CoV S protein seems to have most of its characteristics in common with the S proteins of other coronaviruses, but it will be important for the understanding of the SARS-CoV pathogenic properties to identify the exact conditions of membrane fusion, i.e. pH dependency and protease sensitivity, which can increase the infectivity. The envelope and membrane proteins are integral membrane proteins and required for virus assembly [41]. In the case of the murine coronavirus MHV-A59 the coexpression of the E and M proteins but not the S or N proteins is needed for the release of virus-like particles (VLP) [42]. The nucleocapsid and viral core of the SARS-CoV are likely to be formed by the N protein.

An interesting feature of the SARS-CoV and other coronaviruses is the resistance against the gastrointestinal fluids despite the lipid composition of their envelope. It has been reported that the SARS-CoV can survive in diarrheal stool for four days and also, patients with SARS often suffer from gastrointestinal symptoms with the virus to be detected in the stool [4]. As the molecular basis for the envelope's resistance against acidic environments and gastrointestinal enzymes is unclear, further research has to be carried out in this area which is important for the control of future SARS outbreaks.

### Evolution of the SARS virus

It is unclear when and how novel pathogens such as the SARS-CoV cross the barriers between their natural reservoirs and human populations, leading to the epidemic spread of novel infectious diseases [43]. As with the SARS-CoV, new pathogens are believed to emerge from animal reservoirs and a variety of molecular mechanisms may contribute to the evolution of the viruses or bacteria. Due to the estimated error frequency of 1 × 10 ^-4 ^for RNA-dependent RNA polymerases [44], RNA viruses such as the SARS-CoV can undergo mutation at a high frequency. The SARS-CoV seems to be relatively genetically stable as the RNA sequences from different SARS patients were quite homogeneous. Even the entire genomic sequences of virus isolates from different continental areas did not differ by more than ten amino acids and it seems that two lineages of the virus can be traced [45]. This obvious contradiction to the high potential error rate of the RNA-dependent RNA polymerase suggests the presence of some proofreading mechanism connected with this enzyme. In fact, a detailed analysis of the SARS-CoV genome by bioinformatics indicates the presence of an exonuclease activity [27].

Next to mutations, a further threat of the SARS-CoV is based on the ability of coronaviruses to undergo RNA recombination at a high frequency [15]. For a variety of other coronaviruses, both recombination and mutation in natural infections have been shown to contribute to the diversification of the coronaviruses. Because of the demonstrated ability of coronaviruses to recombinate, the question whether the SARS-CoV will show a higher frequency of mutations within possible future seasonal changes or in respond to drug treatment is an issue of major concern. It was reported that in the initial phases of the SARS epidemic, the mutation rate was high in the gene for the spike protein, but this stabilized during the middle and final stages of the 2003 epidemy [28]. Thus, the virus had experienced great pressure to adapt to the new host after crossing the species barrier, but has then been optimized [28].

### Duration of infection

Although human coronaviruses are characteristically causing self-limiting short diseases, the question of potential chronic SARS infections is of major importance for a future disease control. If the SARS-CoV is able to cause a chronic persistent infection, chronic carriers may serve as sources for new SARS outbreaks. However, the detection of SARS-CoV in stool of patients for longer periods than 6 weeks after hospital discharge has not been reported so far. Therefore, the danger of chronic carriers may not be relevant. In contrast to common human coronavirus infections with short durations, most animal coronaviruses cause persistent infections. As an example, the feline coronavirus FIPV infects animals which then continue to shed virus for periods reaching up to seven months after infection without carrying disease symptoms [46]. Also, TGEV and MHV tend to cause chronic infections as these viruses may be found in the airways and small intestine (TGEV) or the nervous system (MHV) several months after infection [47,48]. Although the SARS-CoV has jumped to humans it may still have this property of inducing chronic infections. Thus, SARS-CoV RNA was found in patients' stool specimen more than 30 days after the infections.

### Clinical picture of SARS

The mean incubation period of SARS was estimated to be 6.4 days (95% confidence interval, 5.2 to 7.7). The mean reported time from the onset of clinical symptoms to the hospital admission varied between three and five days [49].

Main clinical features of the disease are in the initial period common symptoms such as persistent fever, myalgia, chills, dry cough, dizziness, and headache. Further, although less common symptoms are sore throat, sputum production, coryza, vomiting or nausea, and diarrhea [50,51]. Special attention has been paid to the symptom of diarrhea: Watery diarrhea has also been reported in a subgroup of patients one week after the initial symptoms [52].

The clinical course of the disease seems to follow a bi- or triphasic pattern. In the first phase viral replication and an increasing viral load, fever, myalgia, and other systemic symptoms can be found. These symptoms generally improve after a few days. In the second phase representing an immunopathologic imbalance, major clinical findings are oxygen desaturation, a recurrence of fever, and clinical and radiological progression of acute pneumonia. This second phase is concomitant with a fall in the viral load. The majority of patients is known to respond in the second phase to treatment. However, about 20% of patients may progress to the third and critical phase. This phase is characterized by the development of an acute respiratory distress syndrome (ARDS) commonly necessitating mechanical ventilation.

### SARS in adults and children

Rapid progress has been made in understanding the clinical presentation of SARS in adults and children [53-56]. In comparison to adults, SARS seems to be less aggressive in younger children, with no children in one case series requiring supplementary oxygen [57] while in adults, systemic infection as well as respiratory infection may be the rule. SARS is much milder with non-specific cold-like symptoms in children younger than 12 years than it is in adolescents and adults [58]. The reason for the milder clinical presentation of SARS in children is most likely due to differences in developmental stage of the immune system.

The course of the disease in teenagers more likely resembles adults in concerning clinical presentation and disease progression [58]. SARS may also develop severe illness requiring intensive care and assisted ventilation in these adolescent patients. The common presenting features are fever, malaise, coryza, cough, chills or rigor, headache, myalgia, leucopaenia, thrombocytopaenia, lymphopaenia, elevated lactate dehydrogenase levels and mildly prolonged activated partial thromboplastin times [59]. The radiographic findings are non-specific: However, high-resolution computed chest tomography in clinically suspected cases may prove to be an early diagnostic aid when initial chest radiographs appeared normal. While rapid diagnosis with the first-generation RT-PCR assay was not satisfactory, improved RT-PCR assays may help to diagnose SARS in early stages. In this respect, a sensitivity approaching 80% in the first 3 days of illness when performed on nasopharyngeal aspirates may be achieved. The best treatment strategy for SARS among children still has to be determined while no case fatality has been reported in children. In comparison to the prognosis in adults, there is a relatively good short- to medium-term outcome. However, it is crucial to emphasize that continued monitoring for long-term complications due to the disease or its treatment is of major importance [60].

### Molecular mechanisms of SARS virus pathogenesis

#### Cytocidal mechanisms

Coronaviruses are known to exert their effects by cytocidal and immune-mediated mechanisms. In vitro studies using cell culture assays have shown that coronavirus infection commonly results in cytopathic effects such as cellular lysis or apoptosis [61]. Also, the virus can cause cellular fusion leading to the formation of syncytia. These cytopathic effects are caused by steps of the viral replication such as the mobilisation of vesicles to form the viral replication complex [18], leading to the disruption of Golgi complexes [62]. Parallel to results on other coronaviruses, SARS-CoV has been shown to cause cytopathic effects in Vero cells and the formation of syncytia in lung tissues. A further similarity with other coronaviruses seems to be the potential of the SARS-CoV to cause tissue fibrosis [63]. As molecular mechanism for this fibrosis which has been reported for infections with the coronavirus MHV, the N protein has been demonstrated to induce promoter activity of the prothrombinase gene that correlates with fibrin deposition [64].

### Immune-mediated mechanisms

Next to cytocidal effects, also immune-mediated mechanisms of both the innate and adaptive immune system seem to contribute to the pathogenesis of SARS-CoV infections. In this respect, it has been shown that in MHV infection, T cells and cytokines play an important role in development of the disease [65]. Also, humoral antibodies have been reported to be crucial in infections caused by coronaviruses such as FIPV. Herein, antibodies against the spike protein were shown to be related to the induction of peritonitis [66].

For SARS-CoV infections, it has been reported that there seems to be an inflammatory cell influx consisting in particular of macrophages in the airways, and a massive release of cytokines during the peak of the infection [67,68]. It is therefore crucial that these immune mechanisms are further analysed on the molecular level as it seems appropriate that not only antiviral but also anti-inflammatory strategies are evaluated for a use in the clinical management of future SARS cases.

The pharmacotherapy for SARS with anti-inflammatory steroids is controversial and largely anecdotal [69]. It was reported that the initial use of pulse methylprednisolone therapy appears to be more efficacious and equally safe when compared with regimens with lower dosage and should therefore be considered as the preferred steroid regimen in the treatment of SARS, pending data from future randomized controlled trials [70]. A further preliminary, uncontrolled study of patients with SARS, reported that the use of interferon alfacon-1 plus steroids was associated with reduced disease-associated impaired oxygen saturation and more rapid resolution of radiographic lung abnormalities [71].

### Mechanisms of target cell specificity

The most obvious gene which is likely to be a key modifier of SARS pathomechanisms is the spike (S) protein gene. As known for other coronaviruses, it does not only affect viral pathogenesis by determining the target cell specificity but also by other mechanisms. In this respect, a single mutation in the S gene of MHV has significant effects on the viral virulence and tissue tropism [72]. Also, mutations in the S gene led to the emergence of the weakly virulent PRCV from the virulent enteric TGEV [73]. Further potentially important genes are the 'non-essential' ORFs which show a significant divergence between SARS-CoV and other coronaviruses. In this respect, it was reported that the civet cat coronavirus has a 29-nucleotide deletion leading to a fusion of two non-essential ORFs into one new ORF in the SARS-CoV [18]. It was shown that deletion mutants of 'non-essential' ORFs of the group 2 mouse hepatitis virus (MHV) leads to a lower virulence without an impact on viral replication [74]. It has to be established if this also applies to 'non-essential' ORFs of SARS-CoV. Also, other viral gene products such as the M or E proteins may have an impact on the pathogenesis of the disease as they may induce interferon production or apoptosis [75,76].

### Molecular targets for antiviral treatment

The primary target cells of SARS-CoV infection are respiratory epithelial cells. As the virus can also be detected in stool specimen and patients with SARS often also have gastrointestinal symptoms, epithelial cells of the gastrointestinal tract also seem to be major target cells. Next to these epithelial cells, the SARS-CoV has also been found in macrophages and many other cells as it has been detected in not only in the respiratory tract and stool specimen but also in the blood, liver, kidney and urine [6]. In this respect, pathological examination did not only show changes in the respiratory tract, but also in splenic lymphoid tissues and lymph nodes. Furthermore, signs of a systemic vasculitis were found which included edema, localized fibrinoid necrosis, and infiltration of monocytes, lymphocytes, and plasma cells into vessel walls in the heart, lung, liver, kidney, adrenal gland, and the stroma of striated muscles. There was also thrombosis present in veins. Systemic toxic changes included necrosis and degeneration of parenchymal cells of the lung, heart, liver, kidney, and adrenal gland [77]. It may therefore be concluded that SARS can induce a systemic disease and thereby injuring many other organs apart from the respiratory tract.

### Target cell receptors

The SARS-CoV target cell specificity is determined by the spike protein affinity to cellular receptors. In contrast to the all group III coronaviruses and the SARS-CoV for which the receptors have not been finally analyzed, it is known that group I coronaviruses bind to aminopeptidase N (CD13) as receptors [78], while group II coronavirus such as MHV use carcinoembryonic antigen (CEA) as receptor [79].

Recently, it was shown that a metallopeptidase, angiotensin-converting enzyme 2 (ACE2), efficiently binds the S1 domain of the SARS-CoV S protein. SARS-CoV replicated efficiently on ACE2-transfected but not mock-transfected 293T cells. Also, anti-ACE2 but not anti-ACE1 antibodies blocked viral replication on Vero E6 cells, indicating that ACE2 is a functional receptor for SARS-CoV [80] which was also identified by a further study [81].

**Figure 4:**
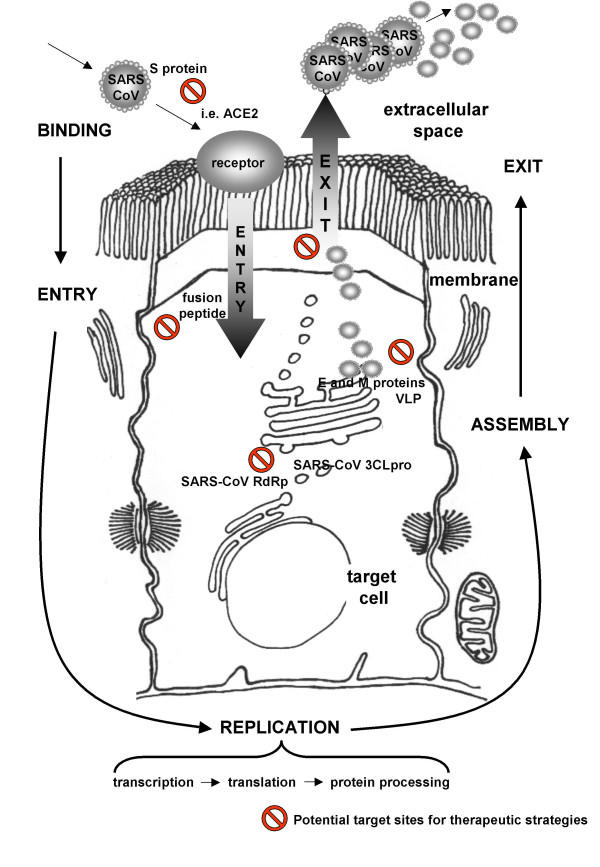
Potential target sites for therapeutic strategies. In view of the viral life cycle, there are several potential targets for the development of antiviral drugs. Starting from the binding of the virus to the target cell, the spike protein or receptors such as angiotension-converting enzyme 2 (ACE2), cell entry or the different replication steps may be targeted. After replication, virus assembly and exit mechanisms may also be used for antiviral strategies. VLP, virus-like particles.

Recently, the C-type lectin CD209L (also called L-SIGN) was discovered to be a further human cellular glycoprotein that can serve as an alternative receptor for SARS-CoV [82]. The interruption of virus-receptor interactions could be a potential target for future therapeutic strategies (Figure 4). In this respect, the receptor-binding S1 domain of the SARS-CoV S protein represents a possible target for new SARS antiviral drugs. Also, antibodies against ACE2, but not inhibitors binding to the active site of ACE2 may be useful for the development of therapeutic strategies.

### Virus entry

After binding to the receptor, the next molecular step of potential use for the development of anti-SARS drugs is the virus entry into the cells. While most coronaviruses enter their target cells via plasma membrane fusion, a further entry mechanism may be acidic pH-dependent endocytosis [83]. Focusing on these mechanisms, it will be crucial to gain further knowledge about SARS-CoV fusion activity. As a drug development candidate, a putative fusion peptide has good potential (Figure 4).

### Intracellular replication

After the binding to a host cell receptor and entry into the cells, the molecular steps of transcription, translation and protein processing display further potential targets for new therapeutic strategies. In this respect, the RNA-dependent RNA polymerases (SARS-CoV RdRp) may be a potential target for a future anti-SARS therapy. A recent study located its conserved motifs and built a three-dimensional model of the catalytic domain [84]. The authors suggested that potential anti-SARS-CoV RdRp nucleotide-analog inhibitors should feature a hydrogen-bonding capability for the 2' and 3' groups of the sugar ring and C3' endo sugar puckering. Also, the absence of a hydrophobic binding pocket for non-nucleoside analog inhibitors similar to those observed in hepatitis C virus RdRp and human immunodeficiency virus type 1 reverse transcriptase seems to be crucial [84].

Also, protease activity is crucial for SARS-CoV RNA replication and protein processing [29,85], and the inhibition of protease function leads to an immediate stop of viral RNA synthesis. Most of the coronaviruses express one major cysteine proteinase, called the main proteinase (M^pro^) or the 3C-like proteinase (3CL^pro^), and two auxiliary, papain-like proteinases (PL1^pro ^and PL2^pro^). The latter two are responsible for the cleavage of the viral polyproteins, pp1a and pp1ab, at three sites near the amino-terminus, while the M^pro ^processes these proteins at as many as 11 additional sites. Interestingly, SARS-CoV lacks the PL1^pro ^[16,17], but it can be assumed that its action is taken over by the PL2^pro ^[29]. This is conceivable since operation of the PL2^pro ^on PL1^pro ^cleavage sites has been shown in IBV and HCoV [86]. Roughly at the position of the PL1^pro ^gene in other coronavirus genomes, SARS-CoV displays a domain within ORF1a that lacks any detectable sequence homology and has therefore been named the SARS-unique domain (SUD) [27]. It is not known whether the SUD protein is ever expressed in the life cycle of SARS-CoV but if it is, it may be connected to the high pathogenicity of SARS-CoV compared to other human coronaviruses and, therefore, it may constitute an attractive target for therapeutic intervention.

Crystal structures have been determined for the M^pro^s of TGEV [87], HCoV 229E [85], and, more recently, SARS-CoV [88]. They all show a similar overall architecture for the 34 kD enzyme which forms a dimer in the crystals and also at intermediate and high concentrations in solution. The monomer consists of three domains of which the first two are β-barrels with an overall similarity to the 3C proteinases of picornaviruses and to the serine proteinase, chymotrypsin. The third domain is α-helical and was shown to be essential for dimerization [85,87,88]. The active site of the enzyme is located in a cleft between domains I and II and comprises a catalytic dyad of Cys...His, rather than the catalytic triad common for cysteine and serine proteinases. Anand et al. [85] have synthesized a substrate-analogous hexapeptidyl chloromethylketone inhibitor and bound it to TGEV M^pro ^in the crystalline state. The X-ray structure of the complex revealed binding of the P1 glutamine, P2 leucine, and P4 threonine side chains of this compound to the respective subsites in the substrate-binding cleft, in agreement with the pronounced specificity for cleavage by the M^pro ^after the substrate sequence (Thr, Val, Ser)-Xaa-Leu-Gln. The structure also showed the expected covalent attachment of the methyl ketone group at P1 of the inhibitor to the catalytic cysteine of the enzyme.

In spite of 40% and 44% sequence identity, respectively, to the M^pro^s of HCoV 229E and TGEV, the crystal structure of the SARS-CoV M^pro ^revealed some surprises [88]. Within the dimer, one molecule was in the active conformation seen in the other structures, whereas the other one adopted a catalytically incompetent conformation. This enzyme had been crystallized at a pH value of <6, which in one of the monomers apparently led to the protonation of a histidine residue at the bottom of the S1 specificity pocket. This resulted in major conformational rearrangements leading to the collapse of this binding site for the P1 glutamine residue of the substrate and to a catalytically incompetent conformation of the oxyanion-binding loop. However, when the crystals were equilibrated at higher pH values, their X-ray structures revealed the active conformation for both monomers in the dimer. This pH-dependent activation mechanism allows interesting conclusions to be made for the self-activation of the M^pro ^from the viral polyprotein, which probably involves a pH-dependent step.

The same hexapeptidyl chloromethylketone inhibitor used by Anand et al. [85] in their crystallographic study of the TGEV M^pro ^was employed by Yang et al. [88] to characterize the interaction of the SARS-CoV enzyme with substrate. This was performed by soaking the inhibitor into crystals grown at the low pH. In spite of the inactive conformation of one of the two monomers in the dimer being preserved, the compound was found to bind to it, but with its P1 glutamine side chain pointing towards bulk solvent rather than into the S1 binding site, because of the collapse of the latter. The binding mode of the inhibitor to the active monomer was also somewhat unusual and is not fully understood at present.

On the basis of their crystallographic work, Anand et al. [85] found that the binding mode of their hexapeptidyl chloromethylketone inhibitor to the TGEV M^pro ^resembled that of AG7088 in complex with its target, the 3C proteinase of human rhinovirus [89], even though the respective target enzymes displayed large structural differences except in the immediate neighbourhood of the active site. AG7088 is in phase II/III clinical studies as an inhalation treatment for the common cold as caused by human rhinovirus. Anand et al. [85] therefore proposed that AG7088 should be a good starting point for the design of anti-SARS drugs, and indeed, the manufacturer of AG7088 confirmed only a few days after their proposal had appeared on-line that the compound was effective against SARS coronavirus in cell culture. AG7088 is now the subject of intensive optimization efforts [90].

Other studies used molecular dynamics simulations of the M^pro ^and screened 29 approved and experimental drugs against a model of the SARS CoV proteinase as well as the experimental structure of the transmissible gastroenteritis virus (TGEV) proteinase [91]. It was suggested that existing HIV-1 protease inhibitors, L-700,417 for instance, may have high binding affinities and may therefore provide another good starting point for the future design of SARS-CoV proteinase inhibitors [92]. However, this has to be proved experimentally.

Further potential targets are the E and M proteins (Figure 4) as they represent the minimum essential components for the assembly of coronaviruses which form the virus-like particles [41,42]. Ultrastructurally, the process of SARS-CoV assembly is most likely localised to the ER-Golgi intermediate compartment [93]. Together with strategies that may focus on the inhibition of virus assembly, the virus exit through secretory pathways is also of interest for the development of new antiviral compounds.

With regard to the multitude of potential epithelial target cells, specific endogenous drug delivery systems may also be of relevance. In this respect, the family of peptide transporters consisting of PEPT1 and PEPT2 which are differentially expressed in potentially infected cells of the respiratory tract [94,95], small intestine [96], kidneys [97,98], nervous system [99] and other organs [100], may serve a target for the rational drug design of antiviral drugs. So far, a variety of antiviral drugs or prodrugs such as valacyclovir [101], valganciclovir [102] or the valyl ester of zidovudine [103] have been shown to be transported via these systems and minimal structure requirements for substrate transport have been determined [104]. A further tool which may be used to approach antiviral therapies is the technique of small interfering RNAs (siRNAs). SiRNAs are double-stranded RNAs which lead to a sequence-specific degradation of mRNAs [105]. Recent in vitro studies used six 21-mer siRNAs that were targeted to different sites of the replicase 1A region of SARS-CoV [106]. Monkey kidney cells (FRhk-4) were infected with the SARS-CoV GZ50 strain and transfected eight hours later with the siRNAs. Three of the six siRNAs led to a marked inhibition of virus cytopathic effects and a reduction of virus copies between 85 and 92 %, indicating that siRNAs may have a potency as antiviral treatment options and that the 1A region displays a promising region to suppress virus replication [106].

### Vaccines against the SARS virus

As most patients develop an immunity against the SARS-CoV and survive the infection, the possibility of creating an effective and safe vaccine seems to exist [107]. There are several options to develop vaccines against the SARS-CoV [108].

### Live-attenuated vaccines

Live-attenuated coronavirus vaccines can be generated by deletions in "group-specific genes". The deletions of these genes do not change replication properties but attenuate the virus [109]. Examples for the use of live-attenuated vaccines to prevent coronavirus infections are live attenuated IBV vaccines which are used in broiler chickens [110]. For the animal coronavirus infections, live attenuated vaccines have been proven to be significantly more effective than whole killed vaccines, indicating that cell-mediated immunity is a crucial defence mechanism. However, the great threat remains that a vaccine strain can recombine with a circulating wild type strain [111] and without evidence that recombination and reversion of a live-attenuated SARS-CoV to virulence can not occur, it is unlikely that a live attenuated SARS-CoV vaccines will be developed and used.

### Whole killed vaccines

Whole killed vaccines are generally safe and easy to generate. In fact, this technique has been applied in veterinary medicine to generate vaccines for BoCV and IBV [112]. Also, an inactivated canine coronavirus vaccine has been produced [113]. A SARS inactivated vaccine was recently developed using the SARS coronavirus (SARS-CoV) strain F69 treated with formaldehyde and mixed with Al(OH)(3) [114]. However, killed vaccines may not protect against different strains of coronaviruses, and live attenuated vaccines have been shown to be more effective than whole killed vaccines in preventing coronavirus animal infections [115].

### Recombinant subunit vaccines

Using molecular biology techniques to generate large quantities of recombinant viral proteins, recombinant subunit vaccines, e.g. against the spike protein, are expected to be created relatively easy as shown by two recent studies [116,117]. Eight recombinant human single-chain variable region fragments (scFvs) against the S1 domain of spike (S) protein of the SARS-CoV from two nonimmune human antibody libraries were screened and one scFv 80R efficiently neutralized SARS-CoV and inhibited syncytia formation between cells expressing the S protein and those expressing the SARS-CoV receptor angiotensin-converting enzyme 2 (ACE2) [117]. A recent study used the SARS-CoV spike protein receptor binding domain (aa 318–510) for immunization, which resulted in the induction of effective neutralizing antibodies [118]. However, recombinant subunit vaccines may have a limited ability to protect against SARS-CoV infections in view of the variations which may arise in the viral genome in future outbreaks. Therefore, the approach of recombinant subunit vaccines may have to be supplemented by further vaccine strategies which focus on cell-mediated immunity.

### Recombinant vectored vaccines

An approach using recombinant vectored vaccines with DNA or a viral vector could be a promising target. The DNA prime and adenovirus or MVA boost approach which is currently analysed for a potential use in the development of HIV vaccines, may also offer a strategy to prevent SARS infections. In this respect, a multi-valent approach which induces both humoral and T cell-mediated host responses seems to be the most attractive strategy.

From the field of veterinary medicine, data on this approach are already available: A recombinant fowlpox with the S1 gene of IBV was demonstrated to be relatively protective against IBV [119]. Also, a DNA vaccine was developed which contains the nucleocapsid protein gene of porcine transmissible gastroenteritis virus (PTGV). This vaccine was shown to initiate both humoral and cell-mediated immune host responses [120]. Recently, three murine studies demonstrated that DNA vaccines encoding different SARS-CoV antigens are capable of generating humoral and cellular immunity and may potentially be useful for control of infection with SARS-CoV [121-123]. However, it was also shown that immunization with modified vaccinia virus Ankara-based recombinant vaccine against SARS is associated with enhanced hepatitis in ferrets [124].

### Epitope-based vaccines

A further strategy is based on the use of epitopes which can be delivered using viral or DNA vectors. Such an epitope-based strategy for coronavirus vaccination has already been reported [125] and the major advantages is the prevention of a possible vaccine reversion to virulence. A further benefit of this technique is the possibility to eliminate any regions of the viral genomic sequence which be associated with a potential autoimmune effects. The limitation of this approach is mainly based on potential variations. In this respect, epitopes which frequently undergo mutations will not protect against the SARS-CoV infections if used in epitope-based vaccines. If the SARS-CoV evolves as a highly variable virus, it will be crucial to identify highly conserved epitopes of the virus.

In summary, the important development of SARS vaccines can be approached using several techniques which should ideally encompass the induction of both humoral and cell-mediated mechanisms. As coronavirus vaccines in animals have partly been reported to cause an enhancement of viral infections [66], a cautious approach has to be followed. A first study has investigated the ability of adenoviral delivery of a codon-optimised SARS-CoV spike protein S1 fragment, membrane protein, and nucleocapsid protein to induce immunity in rhesus macaques. The immunization with a combination of these three Ad5-SARS-CoV vectors and a booster vaccination on day 28 demonstrated antibody responses against the spike protein S1 fragment. Also T-cell responses against the nucleocapsid protein were found and all vaccinated animals displayed strong neutralising antibody responses in vitro. These results indicated that an adenoviral-based vaccine can induce SARS-CoV-specific immune responses in monkeys.

## Conclusion

In summary, the onset of the SARS epidemic in different continents has led to the formation of a successful laboratory network to identify the molecular mechanisms underlying the SARS infection. Next to the development of early diagnostic tests and effective treatment strategies, it is most important to orchestrate research activities which lead to the development of vaccines and antiviral agents, as there is no established therapy to date. Even now in a situation of only a handful of new cases, SARS remains a major global health hazard which may reappear.
